# Living Life on a Magnet

**DOI:** 10.1371/journal.pbio.2000613

**Published:** 2016-08-23

**Authors:** Roland G. Roberts

**Affiliations:** Public Library of Science, Cambridge, United Kingdom

Thanks to a spinning globule of trillions of tons of partially molten iron beneath our feet, living things in most areas of the Earth’s surface are bathed in a geographically asymmetric field of magnetism. We humans appear to be insensitive to magnetic fields, and it took the invention of the magnetic compass to allow us to use the Earth’s magnetic field to help us navigate our way across the planet’s surface. However many other animals have highly developed magnetosensory skills that can enable them to orient themselves in the environment and to migrate over long distances with astonishing accuracy. Bacteria are more interested in the third dimension, and their usual use of magnetotaxis is to help them swim downwards, parallel to the magnetic field lines. Accordingly, bacteria from the Northern and Southern Hemispheres tend to have opposite magnetotactic swimming polarities to cater for the opposing inclination of the magnetic field.

Living things seem to have exploited two principal mechanisms to detect the Earth’s magnetic field. One solution is to use ferromagnetic materials such as magnetite (familiar to our ancestors as lodestone); the other is to use the free radical pairs generated by photoreceptor proteins such as cryptochromes. There is evidence that some organisms may use a combination of these two approaches.

**Fig 1:**
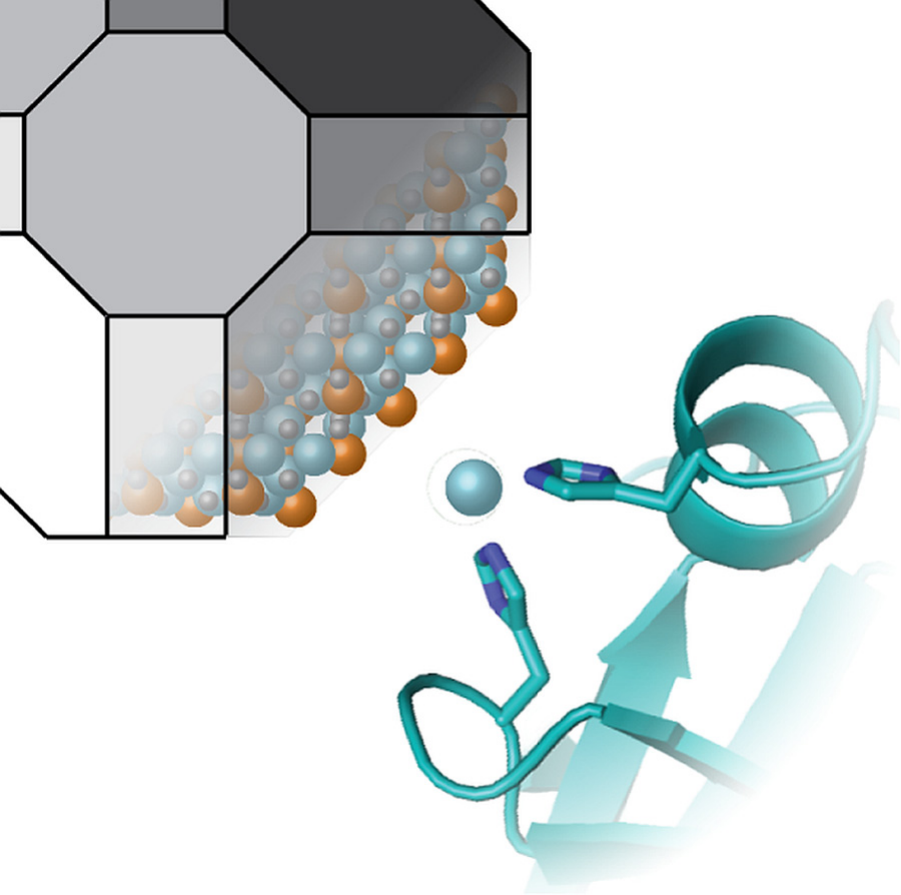
Making magnetite. Two histidines in the protease domain of *M*. *magneticum* MamO (bottom right) bind iron to promote formation of the magnetite crystal (top left). *Image credit*: *doi*:*10*.*1371/journal*.*pbio*.*1002402*.*g008*.

A recent paper in *PLOS Biology* [1] now reveals the molecular details of how magnetotactic bacteria are able to biomineralise iron to make tiny magnetite crystals (Fe_3_O_4_) in compartments called magnetosomes. The authors find that the appropriately named *Magnetospirillum magneticum* has re-purposed an ancient enzyme, once a serine protease from the trypsin family. Rather than snipping proteins, this enzyme–MamO–now uses a pair of histidines to marshal iron ions and contribute to formation of the magnetosome (Fig 1).

*M*. *magneticum* belongs to the α-proteobacteria, and MamO-like genes are widespread in its relatives that share their cubo-octahedral crystals. But magnetotaxis is seen in many branches of the bacterial family tree, and a genetic screen in *PLOS Genetics* [2] identifies some of the genes in the δ-proteobacterium *Desulfovibrio magneticus* involved in making its distinctive tooth-shaped magnetic crystals. Other researchers have used structure-based comparisons of another magnetotaxis gene (*mamA*) shared broadly between magnetotactic bacteria to confirm that this ability arose in a single primordial bacterial ancestor [3], rather than being spread by horizontal gene transfer as some have suggested. A paper published in *Nature Communications* [4] examined bacteria that can switch the polarity of their response to the magnetic field, finding that these bugs (*M*. *gryphiswaldense*) can use a set of chemotaxis proteins to integrate the magnetic cues with independent information about oxygen levels, giving them more nuanced aerotactic abilities.

Like bacteria, nematodes use magnetic cues to direct movement in the vertical axis as they burrow through the soil. A study in *eLife* examined *Caenorhabditis elegans* isolates from around the globe [5], finding that–as with bacteria–worms from the Northern and Southern Hemispheres showed opposing responses to the direction of a magnetic field. They also managed to show that a specific pair of neurons was needed for the worms to navigate appropriately.

By contrast, many animals use magnetism to navigate the horizontal dimensions; a *PLOS ONE* study of Chinese noctule bats [6] found that they tended to cluster towards the magnetic northern end of an enclosure, even when the strength of the magnetic field was reduced to one-fifth of modern levels. The authors argue that this may have allowed the sense to persist through evolution even during periods of magnetic turmoil that have dogged the planet’s history. Turtles also show pronounced magnetotactic alignment, and another *PLOS ONE* study [7] uses the confusion of this sense by radio frequency waves to make a case for a radical pair-based mechanism (perhaps involving cryptochromes) for these animals’ internal compass.

In some cases, loss of magnetosensation can be more serious than mere loss of direction. A further *PLOS ONE* paper [8] finds that removal of the natural magnetic field from a migratory insect, the white-backed planthopper, results in abnormal development, accompanied by disrupted expression of multiple components of the cryptochrome pathway and developmental hormones (cryptochromes are needed to entrain circadian rhythms, which may be therefore affected by the magnetic field). Cryptochromes are again fingered as the smoking gun in the exquisite magnetic perception of birds; a *PLOS ONE* study [9] of the retinas of three migratory bird species–robins, wheatears and pigeons–revealed the intriguing presence of a specific isoform of cryptochrome (Cry1b) in the cytosol of ganglion cells, displaced ganglion cells and rod cell inner segments.

For more detailed reading please see the associated PLOS Collection [10].
